# Authors’ Reply: The SCeiP Model for Remote Rehabilitation in Homebound Patients With Coronary Heart Disease

**DOI:** 10.2196/70247

**Published:** 2025-03-28

**Authors:** Xinyue Zhang

**Affiliations:** 1 Department of Cardiology The First Affiliated Hospital of Nanjing Medical University Nanjing China

**Keywords:** exercise rehabilitation, coronary heart disease, promotion strategy, home rehabilitation

Thank you for the comments on our article, “The Effectiveness of Remote Exercise Rehabilitation Based on the ‘SCeiP’ Model in Homebound Patients With Coronary Heart Disease: Randomized Controlled Trial” [1]. In response to the questions raised by the authors [2], we wish to provide several clarifications. Please refer to the following.

First, considering the individual variations among participants highlighted by the author, we accounted for this factor prior to the study. Consequently, we conducted a specialized statistical analysis of the baseline data of the study participants (T0: week 0) and found no significant differences between the two groups regarding questionnaires and scales, including levels of cardiac rehabilitation cognition, exercise planning, and exercise input. Furthermore, the objective metrics of exercise days and exercise duration were also important outcome indicators.

Second, to evaluate disease severity, 2 primary baseline metrics—New York Heart Association (NYHA) cardiac function classification and the number of diseased vessels—were extracted. Furthermore, other variables such as age and sex were analyzed in the baseline data and found to have no significant differences, thus minimizing their potential influence on the results.

Other unexplored areas will be addressed in future research. We are grateful for the insightful comments provided by Zhang and Chen [2] on our article.

## Abbreviations

NYHA: New York Heart Association
